# Observations on the Cure of Acute Rheumatism

**Published:** 1814-02

**Authors:** C. E. Lucas

**Affiliations:** Hatfield


### Medical and Physical Journal.

2 OF VOL. XXXI.] FEBRUARY, 1814. [NO. 180.

"For many fortunate discoveries in medicine, and for the detection of nume-
"rous errors, the world is indebted to the rapid circulation of Monthly
"Journals; and there never existed any work to which the faculty in
"EUROPE and AMERICA were under deeper obligations than to the
"Medical and Physical Journal of London, now forming a long, but an
"invaluable series."—RUSH.

*For the Medical and Physical Journal.*

### *Observations on the Cure of Acute Rheumatism; by* Mr. C. E. LUCAS, of Hatfield.

THE observations of Dr. Sutton on Acute Rheumatism, in No. 177 of the Medical and Physical Journal, are valuable, not only as recommending a successful mode of practice, but as establishing it on rational principles. The use of cold applications externally, must, however, wait the test of a more enlarged experience to determine either its efficacy or safety as a remedy. I have had no experience of its effects, but shall be extremely happy to find that it succeeds, as it would undoubtedly prove a most desirable acquisition to our curative means. It is however not, I conceive, likely to supersede the necessity for the other parts of the antiphlogistic plan of treatment, but rather to prove an useful auxiliary, as judiciously recommended by Dr. S.

The strictures of Dr. S. on the bark in this disease, as recommended by Dr. Haygarth, would lead us to expect little or nothing from it as a remedy; and he accounts for its supposed efficacy, by observing that cases of the disease will be very short under various modes of treatment. This is not, I think, doing justice to a remedy, which, under certain regulations, is frequently beneficial; and indeed the treatment recommended by Dr. S., with the exception of the cold lotions, still "sub judice," is by no means so generally successful in removing this disease, as to induce us hastily to abandon a remedy of promise.

I have used the bark very freely in this disease, and, as I think, with the most beneficial effects, though generally in conjunction with either henbane or opium, and never until some means had been taken to diminish vascular excitement.

The henbane has appeared to me a most valuable remedy in this disease, and I attribute the success I have had in the speedy speedy cure of several of the worst cases of acute rheumatism to this medicine more than to any other. One of the most severe cases of the disease that I ever met with, yielded in a week to a bleeding of 18 oz., a dose of antimonial powder and calomel at night, with a dose of physic next morning, followed by $2\frac{1}{2}$ grs. of the extract of henbane every four hours, with 12 grs. of nitre. I am the more disposed to ascribe much efficacy to the henbane in this disease, from witnessing its general happy effects in other cases of increased vascular excitement attended with morbid sensibility, particularly in nettle rash, in which I regard it almost as a specific.

The plan I generally follow in acute rheumatism, is to bleed to 18 or 20 oz., to give the calomel and antimonial powder in full doses the two first nights, with the magnes. vitriolat. in the mornings, and afterwards to direct the henbane in doses of from 3 to 6 grs. every four or six hours. If this plan should not succeed in a few days, I add the bark in powder, in doses of ʒj every four hours, and should then direct an opiate at night. By these means I have generally succeeded in removing the most violent attacks of this disorder in the course of a fortnight, and often in half the time. If the disease under this treatment should not give way in about a week, I interpose a brisk purgative, and resume the bark in full doses with the opiate at night, but without the henbane. And in all cases where the disease has been of long standing, my chief reliance is on the bark.

It is always difficult, if not impossible, to point out the modus operandi of medicines; and it is perhaps sufficient for practical purposes to ascertain their effects, so as to found their employ upon rational and consistent principles. That the bark has the property of relieving intermittent pains, will hardly be denied; that disorders in which distinct intermissions occur, are generally connected more or less with atony of the system, seems also established; that the bark relieves intermittent pains, by counteracting that atony upon which is founded the morbid sensibility of nerves, is probable; but whether it has any property of relieving other pain, when by previous treatment the same condition of parts may be supposed to prevail, must be doubtful; but I incline to think that this may be the case. At all events it seems generally agreed that the bark can in no case, either with or without pain, be given with propriety, whilst there is any considerable inflammatory diathesis present.

*Hatfield,*

*Dec.* 14, 1813.

